# Decoding Surface-Bound Isomers: A QIT-ToF-SIMS Platform Integrating Tandem Mass Spectrometry and Laser Spectroscopy

**DOI:** 10.1021/acs.analchem.5c05564

**Published:** 2025-11-06

**Authors:** Ji Young Baek, Jae Yeong Eo, Eombae Moon, Myoung Choul Choi, Chang Min Choi

**Affiliations:** † Center for Scientific Instrumentation, Division of R&D Equipment Industry, 65398 Korea Basic Science Institute, Daejeon 34133, Republic of Korea; ‡ Division of R&D Equipment Industry, Korea Basic Science Institute, Daejeon 34133, Republic of Korea

## Abstract

A quadrupole-ion-trap-integrated time-of-flight secondary ion mass spectrometry (QIT-ToF-SIMS) system was developed to enable detailed molecular surface analysis via tandem mass spectrometry combined with laser photodissociation spectroscopy. In this approach, secondary molecular ions were selectively isolated in the ion trap using the stored waveform inverse Fourier-transform technique and subsequently subjected to collision-induced dissociation or photoinduced dissociation. Using isomeric rhodamine dyes as model systems, structurally diagnostic fragment ions were observed, allowing for clear molecular identification despite identical nominal masses. The laser photodissociation spectra obtained for the isolated ions revealed a distinct wavelength-dependent fragmentation behavior, consistent with known gas-phase absorption profiles. Because of the integration of tandem mass spectrometry and laser spectroscopy with time-of-flight secondary ion mass spectrometry, the QIT-ToF-SIMS system serves as an effective tool for molecular-level surface characterization, which is particularly well suited for probing increasingly complex organic materials and interfaces.

## Graphical abstract

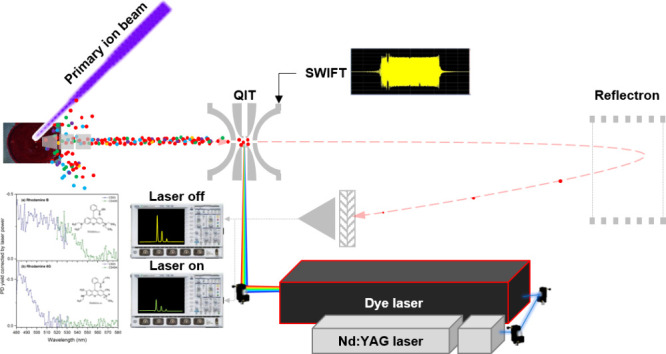

## Introduction

In recent years, surface mass spectrometry (MS) has gained widespread attention due to the increasing demand for molecular-level analysis in interfacial chemistry, advanced material surface characterization, and biological systems under realistic conditions.^1–4^ Representative ionization techniques for surface MS include matrix-assisted laser desorption ionization,^5^ desorption electrospray ionization,^6^ and secondary ion mass spectrometry (SIMS)^7–10^ using primary ion bombardment. The choice of ionization technique and mass spectrometer depends on the analyte properties, the size (or molecular weight) of the analyte, and the required resolution for image analysis.^1^ Among the various imaging MS techniques reported to date, SIMS offers the highest spatial resolution and allows for the depth profiling of multilayered samples, thereby enabling a form of three-dimensional analysis. This characteristic renders SIMS one of the most powerful techniques currently available for surface characterization.

Time-of-flight secondary ion mass spectrometry (ToF-SIMS) is a continuously evolving and widely used tool for surface analysis. To enhance the detection of intact molecular ions using this technique, various primary cluster ion beams have been developed. Compared to high-energy atomic ion beams, large cluster ions distribute the impact energy across multiple atoms, resulting in gentler sputtering and enhanced molecular ion yields.^11,12^ This advancement has significantly broadened the application scope of ToF-SIMS, particularly in fields such as bioanalysis and organic electronics.^13,14^ Consequently, the development of new, larger cluster ion beams to improve the efficiency of secondary ion detection remains an active area of research. For example, water cluster ion beams have been developed using water vapor^15^ and the vacuum electrospray droplet impact method^16,17^ to enhance the ionization efficiencies of biological samples. Additionally, an organic molecular cluster ion beam, generated via multiphoton ionization, was developed to observe an intact organic molecular secondary ion.^18^ Furthermore, mixed gas cluster ion beams have been proposed to further improve the ionization efficiency and beam control.^19^ These innovations collectively represent a major trend in SIMS development, i.e., the enhancement of secondary molecular ion detection via novel primary ion beam design.

The combination of ToF-SIMS with these cluster ion beams has extended the scope of SIMS from materials science to biological applications.^20^ Time-of-flight mass spectrometry (ToF-MS) offers a range of advantages, including rapid analysis, reduced levels of sample damage, and a theoretically unlimited mass range with simultaneous detection capabilities. However, despite these advantages, ToF-MS suffers from a limited mass resolving power due to its inherent physical principles. Additionally, many cluster ion sources are unable to produce sufficiently short pulses, thereby limiting the efficiency of secondary ion bunching. As a result, the peak assignment becomes challenging when multiple isobaric species are present. Moreover, structural isomers that share identical nominal masses remain difficult to distinguish, particularly when fragment information is inaccessible.

To address these challenges, tandem mass spectrometry (MS/MS) has been introduced into the ToF-SIMS systems. Indeed, several research efforts have attempted to integrate high-resolution analyzers, such as Orbitraps, with ToF-SIMS, to achieve an enhanced mass resolution and promote molecular identification via collision-induced dissociation (CID).^21,22^ A triple-focus electrostatic analyzer combined with an additional ToF-MS module has also been developed for the MS/MS of secondary ions.^23^ However, these systems are often limited in terms of their flexibility, in addition to requiring substantial modifications or lacking the capability to interface seamlessly with selective ion isolation and photodissociation strategies. Thus, despite recent advances in surface MS, it remains necessary to develop techniques that can simultaneously provide molecular selectivity, structural identification, and spectroscopic insight, especially for complex isomeric mixtures on heterogeneous surfaces.

In this study, a newly developed quadrupole ion trap (QIT)-integrated ToF-SIMS (QIT-ToF-SIMS) instrument capable of selective secondary molecular ion isolation and MS/MS is presented. The integration of a QIT allows for the storage and isolation of specific molecular ions using the stored waveform inverse Fourier transform (SWIFT) technique. These isolated ions are then subjected to laser photodissociation, and the resulting fragment ions are analyzed by using a reflectron ToF-MS device. Compared with previous tandem SIMS approaches, this QIT-ToF-SIMS permits highly flexible ion selection, efficient isolation via tailored waveforms, and direct coupling to photodissociation analysis, all within a compact and modular architecture. This combined system enables structural elucidation and isomer discrimination directly at the sample surface, offering new analytical capabilities at the interface between MS and laser spectroscopy. Such integration is particularly valuable for studying biologically relevant molecular systems, organic coatings, and soft materials where conventional MS workflows often fail to resolve the structural ambiguity. In summary, this work uniquely demonstrates how SIMS-based molecular analysis can be extended beyond conventional limits through the seamless integration of selective ion trapping, tandem MS, and laser spectroscopy. To the best of our knowledge, such an analytical capability—enabling both isomer discrimination and wavelength-resolved photodissociation of surface-bound ions—has not been previously reported.

## Experimental Section

The experimental apparatus used in this study was a custom-built QIT-ToF-SIMS instrument, as shown schematically in Figure 1. The primary ion source was an organic molecular cluster ion beam, which has been described in detail elsewhere.^18^ A brief summary of the setup, procedures, and modifications relevant to this study are provided herein.

**Figure 1:**
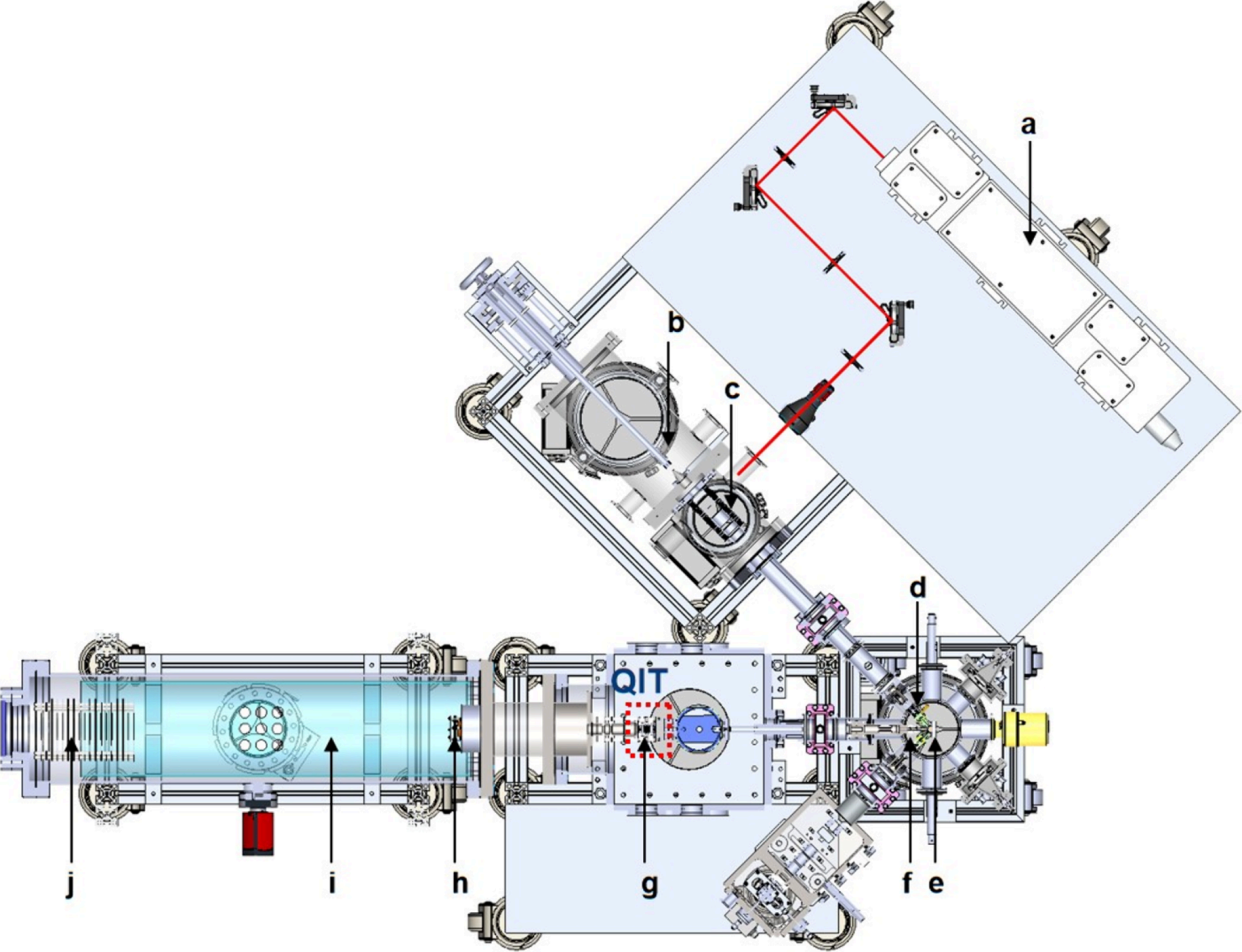
Schematic representation of the QIT-ToF-SIMS instrument combining a cluster ion source, QIT, and ReToF-MS: (a) EKSPLA NL204–1K laser, (b) OCIB nozzle, (c) ion beam optics, (d) objective lens set, (e) four-axis sample manipulator, (f) extraction lens for secondary ions, (g) QIT, (h) MCP dual detector assay, (i) flight tube liner/shield, and (j) reflector assay.

To generate the cluster ion beam, toluene vapor was coexpanded with argon gas through a continuous nozzle (Figure 1b), forming a supersonic molecular beam. The toluene molecules and clusters were ionized via multiphoton ionization using the fourth harmonic (266 nm) of a Nd:YAG laser (0.6 mJ/pulse, pulse duration = 7–10 ns, repetition rate = 1 kHz; NL204-FH, EKSPLA, Lithuania). The resulting toluene cluster ions were accelerated by using a potential of 20 kV and focused through a cylindrical lens assembly (Figure 1c). To isolate only the toluene monomer ions (*m*/*z* 92), a mass gate employing a negative high-voltage pulse (1.0 kV) was used. These selected ions were further focused using an objective lens system (Figure 1d) and directed toward the sample stage (Figure 1e) to generate secondary ions by 20 keV bombardment. Rhodamine 6G and Rhodamine B (Sigma-Aldrich, St. Louis, USA) were used without purification and were dissolved in ethanol to prepare 1 mM stock solutions. Each solution was drop-cast onto a stainless steel plate (6 × 6 mm, 1 mm thick) to form thin molecular films of the respective rhodamine derivatives. In addition, a commercial red ink pen (Monami, Republic of Korea) was directly applied to a stainless steel plate to prepare a third sample, which was used to evaluate the MS/MS capability of the system. All three prepared samples were mounted on the sample stage simultaneously for analysis.

The secondary ions sputtered by toluene ion bombardment (20 keV) were accelerated toward the QIT (Figure 1g) by applying an extraction pulse of approximately +80 V. After stably storing the secondary ions in the QIT through two sets of lenses, the SWIFT pulses were applied to the exit end-cap of the QIT to isolate the target molecular ions from the other residual ions. The SWIFT pulses were generated using an arbitrary function generator (AFG 3022, Tektronix) using the algorithm developed by Doroshenko and Cotter.^24^ Subsequently, CID or photoinduced dissociation was performed by introducing the collision gas or irradiating with a laser pulse, respectively. The positive pulse was applied to the entrance end-cap electrode to extract all ions inside the QIT into the ToF-MS. The extracted ions were accelerated into the field-free drift region of a reflectron ToF-MS instrument (ReToF-MS, R.M. Jordan Co.) The ion signals from the microchannel plate (MCP, Figure 1h) were processed using a 500 MHz digital storage oscilloscope (LT374, LeCroy).

To investigate the structural and optical properties of the selectively stored secondary molecular ions in the QIT, laser-induced spectroscopy was performed. The QIT was irradiated with the output of a dye laser pumped by the third harmonic (355 nm) of a Nd:YAG laser. The dye laser was scanned from 480 to 580 nm in 1 nm increments using Coumarin 540A (for wavelengths of 480–530 nm) and Coumarin 503 (for wavelengths of 520–580 nm), thereby covering the visible range relevant to the rhodamine derivatives. A positive direct current pulse was applied to the entrance end-cap electrode immediately after irradiation to extract all ions from the QIT into the ReToF-MS. The extracted ions were reflected by the ion mirror and detected using an MCP detector. Photodepletion spectra were obtained by recording the depletion yields of the parent ions as a function of the laser wavelength. Since the intensities of the secondary molecular ions generated by the primary ion beam may fluctuate over time, normalization was essential to account for such variations. Specifically, to minimize the influence of the ion signal instability on the photodepletion spectra, the 20 Hz acquisition rate of the mass spectrometer was divided into two synchronized channels. Odd-numbered pulses were synchronized with the 10 Hz repetition rate of the laser system, while even-numbered pulses were recorded without laser irradiation (Figure S1). The mass spectra obtained from each channel were stored separately, and the photodepletion yield at each wavelength was calculated using the following equation:

$$PDyield=\frac{I_{off}-I_{on}}{I_{off}}\times \frac{1}{\lambda E}$$

where *I*_*off*_ and *I*_*on*_ are the integrated signal intensities of the parent ion in the absence and presence of the laser, respectively, while λ is the laser wavelength, and E is the pulse energy. This normalization compensates for variations in the photon flux at each wavelength. This alternating acquisition and normalization scheme minimizes spectral artifacts caused by long-term signal drift during extended measurement times.

## Results and Discussion

The capability of the QIT-ToF-SIMS system to selectively isolate target ions using the SWIFT technique was demonstrated using Rhodamine 6G as a model analyte. Figure 2 compares the secondary ion mass spectra obtained with and without application of the SWIFT waveform. In the absence of the SWIFT waveform (Figure 2a), all secondary ions generated by the primary ion beam were stored in the QIT and transferred to the ToF-MS instrument. The resulting spectrum displayed a complex array of peaks, including numerous fragment ions and chemical background signals, with the intact molecular ion of Rhodamine 6G being observed at *m*/*z* 443. This extensive fragmentation is a well-known characteristic of SIMS when high-energy primary ion beams are employed because these beams deposit substantial energy onto the surface and often lead to rich but convoluted spectra. While such fragmentation can be beneficial for surface-sensitive structural analysis, it often complicates spectral interpretation and obscures the assignment of specific molecular species. This inherent limitation highlights the necessity of MS/MS in SIMS-based systems, especially when dealing with isobaric interferences or structurally similar compounds. Without precursor ion isolation, it becomes challenging to confidently identify target molecules within complex matrices. Upon application of the SWIFT waveform (Figure 2b), all ions, except that at *m*/*z* 443, were selectively removed from the trap, resulting in a highly purified spectrum consisting solely of the target molecular ion peak. This clean isolation confirms the high selectivity and resolution of SWIFT-based ion manipulation in the QIT. Such selective ion storage is essential for MS/MS and laser spectroscopy experiments as it ensures that only the desired precursor ion contributes to the observed fragmentation or photodissociation pattern. Together, these results underscore the value of incorporating precursor ion isolation techniques, such as SWIFT, into SIMS instruments, ultimately enabling more accurate and confident structural elucidation in complex samples.

**Figure 2:**
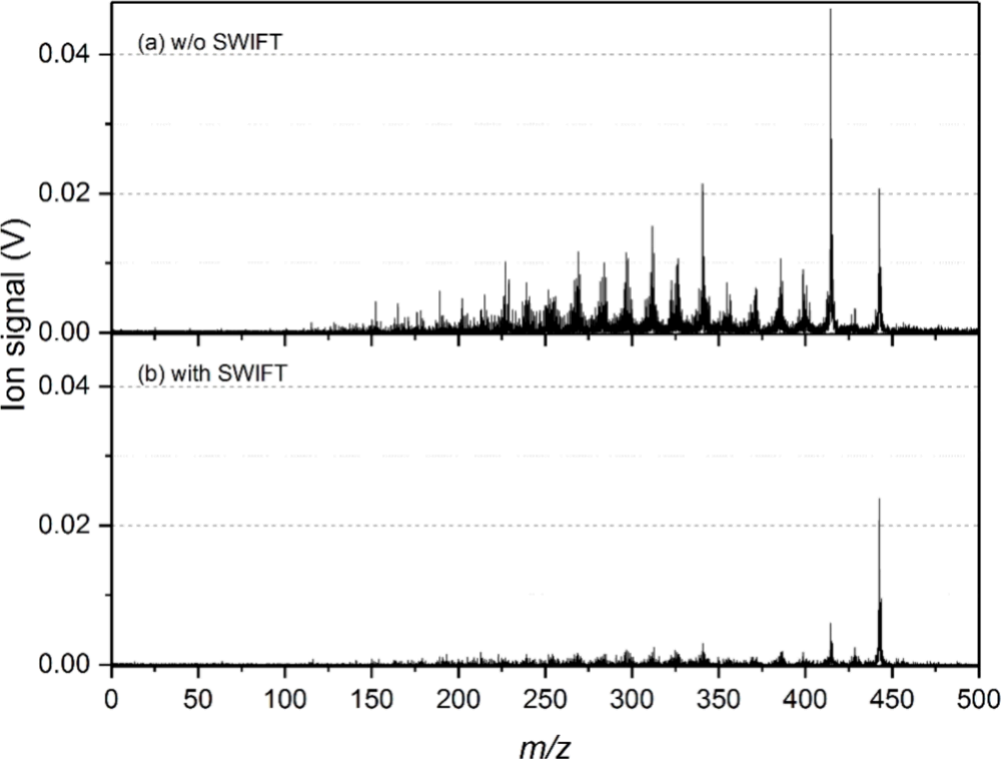
Secondary ion mass spectra of Rhodamine 6G acquired using the QIT-ToF-SIMS system: (a) Without the application of SWIFT (i.e., all secondary ions stored), and (b) with application of the SWIFT waveform to selectively isolate the *m*/*z* 443 ion in the QIT. In the absence of SWIFT, a wide distribution of fragment and background ions is observed. Upon the application of SWIFT, only the targeted intact molecular ion at *m*/*z* 443 remains, confirming the high ion selectivity of the isolation process.

To evaluate the MS/MS capability of the developed QIT-ToF-SIMS instrument, we selectively isolated the *m*/*z* 443 secondary ions, which correspond to the intact molecular forms of Rhodamine B and Rhodamine 6G, using the SWIFT technique in the QIT. These two compounds were chosen because they share the same nominal mass yet differ structurally, allowing us to highlight the tandem MS and laser spectroscopy capabilities of the system in distinguishing isomeric surface species. CID was subsequently performed within the trap, and the resulting fragment ions were analyzed by ReToF-MS. Figure 3 shows the CID spectra obtained from thin films prepared using Rhodamine B, Rhodamine 6G, and a commercial red ink. In each case, only the *m*/*z* 443 ion was stored in the QIT prior to fragmentation, ensuring high specificity for MS/MS. The CID spectra clearly differentiate between the two isomeric rhodamine compounds, despite their identical molecular masses. Rhodamine B (Figure 3a) shows a dominant fragment ion at *m*/*z* 399, corresponding to the neutral loss of a carboxyphenyl group. In contrast, Rhodamine 6G produces a main fragment ion at *m*/*z* 415, which arises from the cleavage of its ethylamino substituent. These characteristic fragmentation patterns serve as structural fingerprints that allow for confident molecular identification. Additionally, the CID spectrum of the red ink sample (Figure 3b) displays a strong fragment ion at *m*/*z* 415, closely matching the dominant fragment observed for Rhodamine 6G. This result suggests that Rhodamine 6G, rather than Rhodamine B, is the major chromophore present in the red ink. Such identification would not be feasible based on the molecular ion alone, underscoring the power of MS/MS for isomer discrimination. Notably, the red-colored spectra overlaid in each panel were adapted from a previous study^25^ and serve as reference CID profiles for Rhodamine B and Rhodamine 6G. This excellent agreement between the main fragment ions (*m*/*z* 399 for Rhodamine B and *m*/*z* 415 for Rhodamine 6G) validates the accuracy of the current measurement approach. More importantly, these results demonstrate that fragmentation patterns can serve as reliable identifiers for molecular structures, enabling confident identification, even in unknown samples. Such spectral matching is especially valuable for practical applications at the molecular level, including forensic analysis and surface characterization.

**Figure 3:**
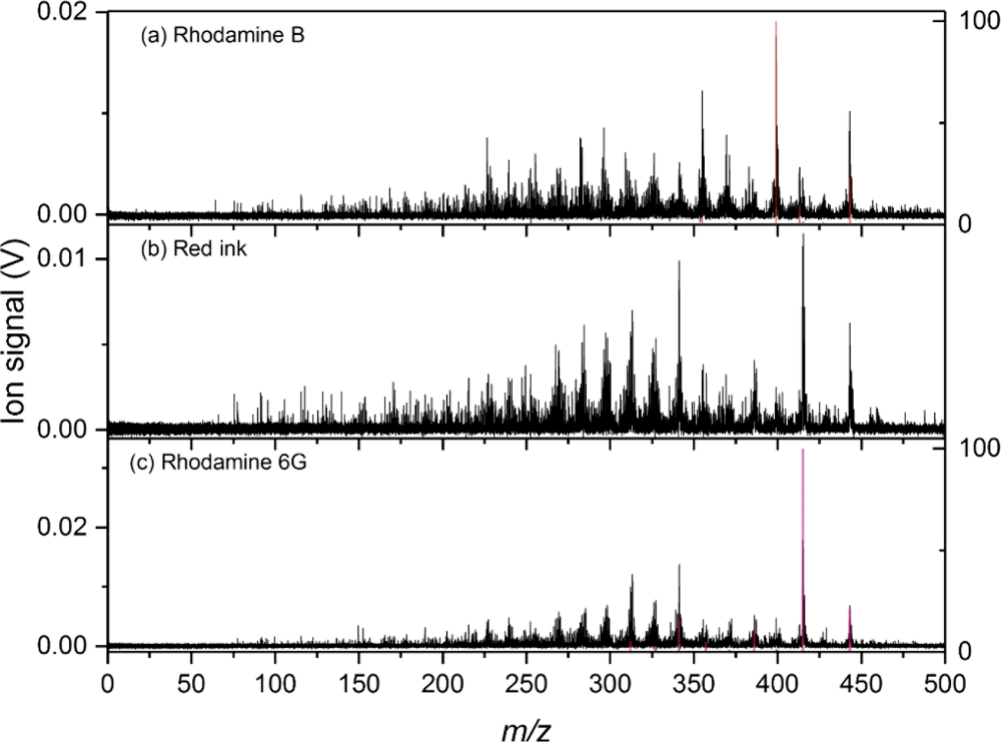
Secondary ion mass spectra obtained from (a) Rhodamine B, (b) a commercial red ink, and (c) Rhodamine 6G thin films measured using the QIT-ToF-SIMS instrument. For comparison, the red-colored spectra overlaid in each panel were adapted from the literature.^22^

The laser-induced photodissociation behavior of the selected secondary ions was initially explored at discrete wavelengths using differential mass spectra (Figures S2 and S3). For both Rhodamine B and Rhodamine 6G, laser irradiation at specific wavelengths led to clear photofragment ion formation along with photodepletion of the parent ion (*m*/*z* 443), confirming that photoinduced fragmentation could be achieved in a wavelength-dependent manner. Building upon this, continuous wavelength scans were performed using two dyes (Coumarin 503 and Coumarin 540A) to obtain detailed photodepletion spectra over the wavelength range of 480–580 nm. The raw photodepletion yields and corresponding laser power curves are provided in Figures S4 and S5. To account for fluctuations in the laser output power, the photodepletion yields were normalized based on the laser power at each wavelength. More specifically, Figure 4 presents the normalized photodepletion spectra recorded for Rhodamine B and Rhodamine 6G, highlighting their distinct optical absorption behaviors in the gas phase. Rhodamine B exhibits a broad absorption profile with significant photodissociation below 550 nm, while Rhodamine 6G shows strong photodepletion only below 520 nm and exhibits negligible absorption beyond 540 nm. These trends are consistent with the molecular structures and conjugation patterns of the two xanthene derivatives. Notably, the observed wavelength-dependent photodepletion spectra are in excellent agreement with previously reported gas-phase photofragmentation spectra for xanthene-based dyes,^26^ further validating the structural fidelity of the isolated ions and the utility of the present spectroscopic approach.

**Figure 4:**
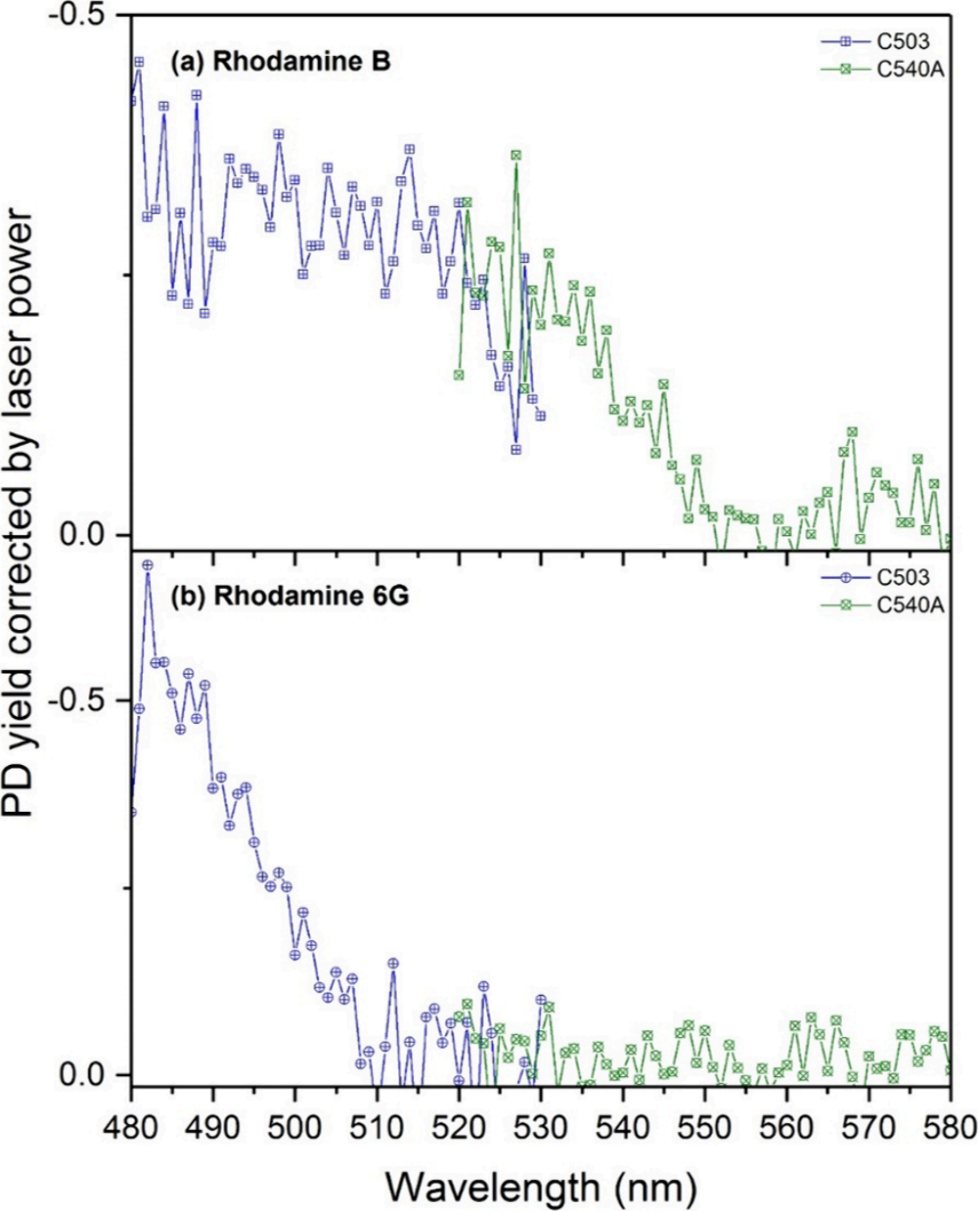
Power-normalized photodepletion spectra of (a) Rhodamine B and (b) Rhodamine 6G measured by using two dye laser systems (Coumarin 503 and Coumarin 540A). The photodepletion yield at each wavelength was corrected by the corresponding laser power to reveal the intrinsic photodissociation behavior.

Overall, the combined results from selective ion isolation, MS/MS, and laser spectroscopy clearly demonstrate the analytical versatility of the developed QIT-ToF-SIMS instrument. By enabling both structural elucidation and optical characterization, this system provides a powerful platform for analyzing complex samples, identifying unknown compounds, and exploring molecular isomerism directly on surfaces. Importantly, beyond this versatility, the instrumental integration allows not only conventional ToF-SIMS analysis but also tandem mass spectrometry and laser spectroscopy to be performed within a single platform, offering a practical solution to long-standing analytical challenges in air-sensitive materials and advanced surface characterization that have been difficult to address with existing techniques.

## Conclusion

A QIT-ToF-SIMS system was developed for the combined application of MS/MS and laser spectroscopy, enabling the enhanced structural and optical characterization of surface-bound molecular species. Additionally, selective precursor ion isolation was achieved using the SWIFT technique, allowing for the unambiguous fragmentation analysis of isomeric compounds via CID. Using Rhodamine B and Rhodamine 6G as model systems, the instrument effectively distinguished between structurally similar species with identical nominal masses, underscoring the analytical strength of fragmentation fingerprinting for molecular identification. Laser-induced photodissociation spectroscopy was also implemented, providing direct access to the wavelength-dependent photodepletion spectra of the isolated secondary ions. The resulting photodepletion spectra closely matched known photophysical behaviors, thereby validating the spectroscopic accuracy and molecular integrity preserved through the ion isolation process. Through this combination of molecular ion isolation, MS/MS, and optical spectroscopy in a compact SIMS platform, the developed QIT-ToF-SIMS system presents a versatile and high-precision tool for surface analysis. This technological integration is poised to advance the frontiers of molecular-level surface science, offering new capabilities for the exploration of complex organic interfaces, biomolecular assemblies, and light-responsive materials, thereby laying the groundwork for the next generation of functional surface diagnostics. By bridging advanced ion manipulation with in situ spectroscopic interrogation, this integrated platform offers a paradigm shift in surface-specific molecular analysis, delivering mechanistic insights and molecular resolution that are highly sought after in modern chemical science. Furthermore, by integration with the recently developed water droplet ion beam, which contains a high abundance of protons, this system will be expected to enable MS/MS and laser spectroscopy to be performed for protonated secondary peptide ions that have been directly sputtered from biological samples. This should offer a powerful new tool for elucidating their structural, photophysical, and photochemical properties.

## Supplementary Material



## Notes

Additional experimental details, instrumental schematics of the QIT-ToF-SIMS system, MS/MS spectra of Rhodamine B and Rhodamine 6G, laser photodissociation spectra, and supporting Figures S1–S5 (PDF)
