# Supplementary material for: Decoding Surface-Bound Isomers: A QIT-ToF-SIMS Platform Integrating Tandem Mass Spectrometry and Laser Spectroscopy
Source: Anal Chem. 2025 Nov 6;97(46):25797–802. doi: 10.1021/acs.analchem.5c05564 (PMC12659776; doi:10.1021/acs.analchem.5c05564)
Supplement: Supplementary file 1 [file ac5c05564_si_001.pdf]

Supporting Information

Decoding Surface-Bound Isomers: A QIT-ToF-SIMS Platform Integrating Tandem Mass Spectrometry and Laser Spectroscopy

Ji Young Baek,[a] Jae Yeong Eo,[b] Eombae Moon,[b] Myoung Choul Choi[a] and Chang Min Choi[b,]*

[a]Center for Scientific Instrumentation, Division of R&D Equipment Industry, Korea Basic Science Institute, Cheongju, Republic of Korea.

[b]Division of R&D Equipment Industry, Korea Basic Science Institute, Cheongju, Republic of Korea

Table of Contents

Figure S1. Schematic of the timing and synchronization scheme for the photo-induced dissociation experiments performed using the QIT-ToF-SIMS system S3

Figure S2. Differential mass spectra of Rhodamine B upon laser irradiation at various wavelengths (480, 500, 520, and 540 nm) …………………………………...S4

Figure S3. Differential mass spectra of Rhodamine 6G obtained upon laser irradiation at 520 and 540 nm ………………………………………………………………..…S5

Figure S4. Photodepletion spectrum recorded for Rhodamine B across the 480–580 nm wavelength range using two laser dyes, namely Coumarin 503 and Coumarin 540A ……………………………….………………………………………S6

Figure S5. Photodepletion spectrum recorded for Rhodamine 6G across the 480–580 nm wavelength range using two laser dyes, namely Coumarin 503 and Coumarin 540A ………………………………………………………………….………S7

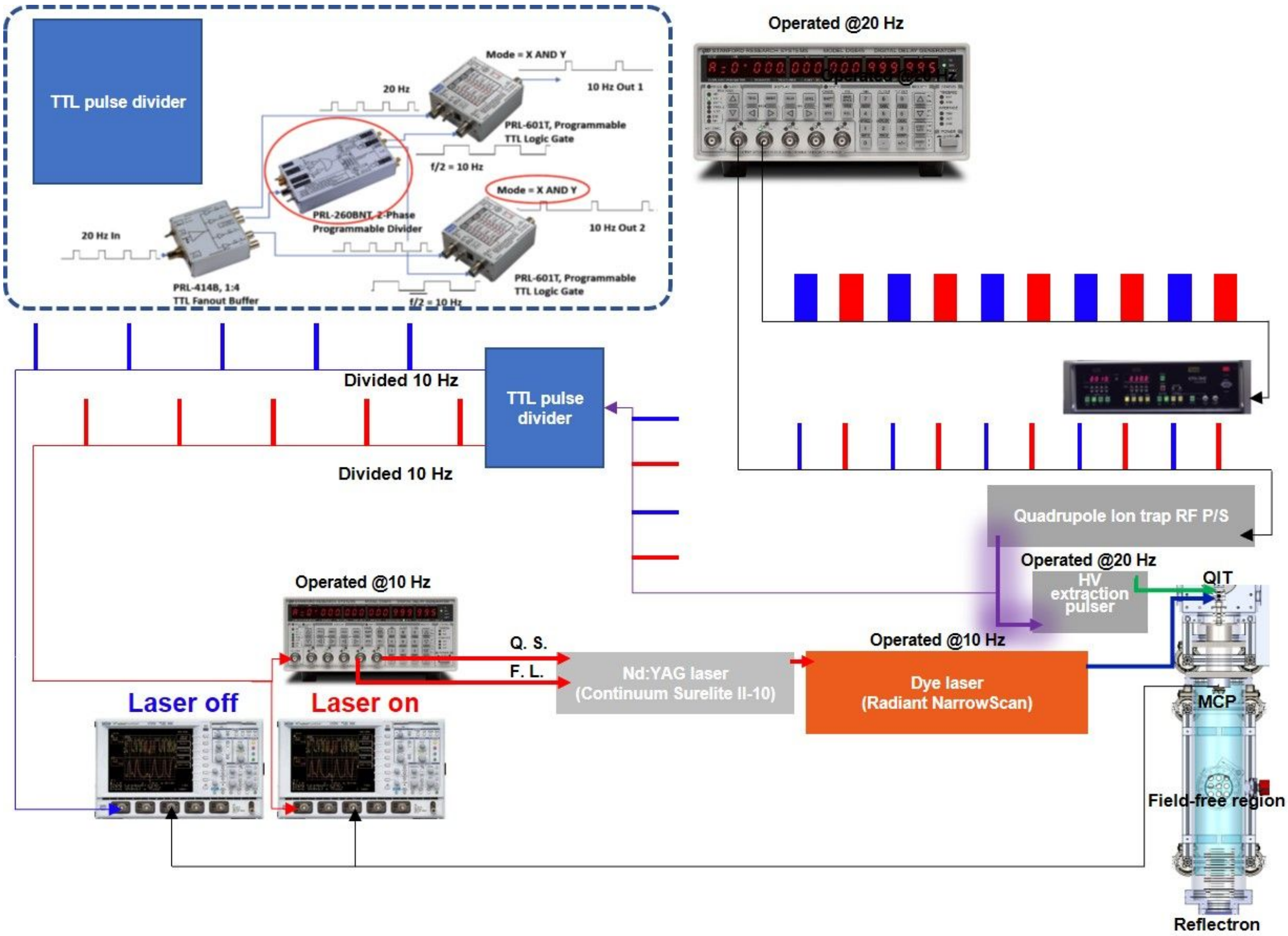

Figure S1. Schematic of the timing and synchronization scheme for the photo-induced dissociation experiments performed using the QIT-ToF-SIMS system. A 20 Hz TTL master pulse was divided into 10 Hz alternating pulses using programmable logic gates and pulse dividers. Odd-numbered pulses (red) were synchronized with the Nd:YAG-pumped dye laser (Radiant NarrowScan) for laser-on measurements, while the even-numbered pulses (blue) correspond to the laser-off cycles.

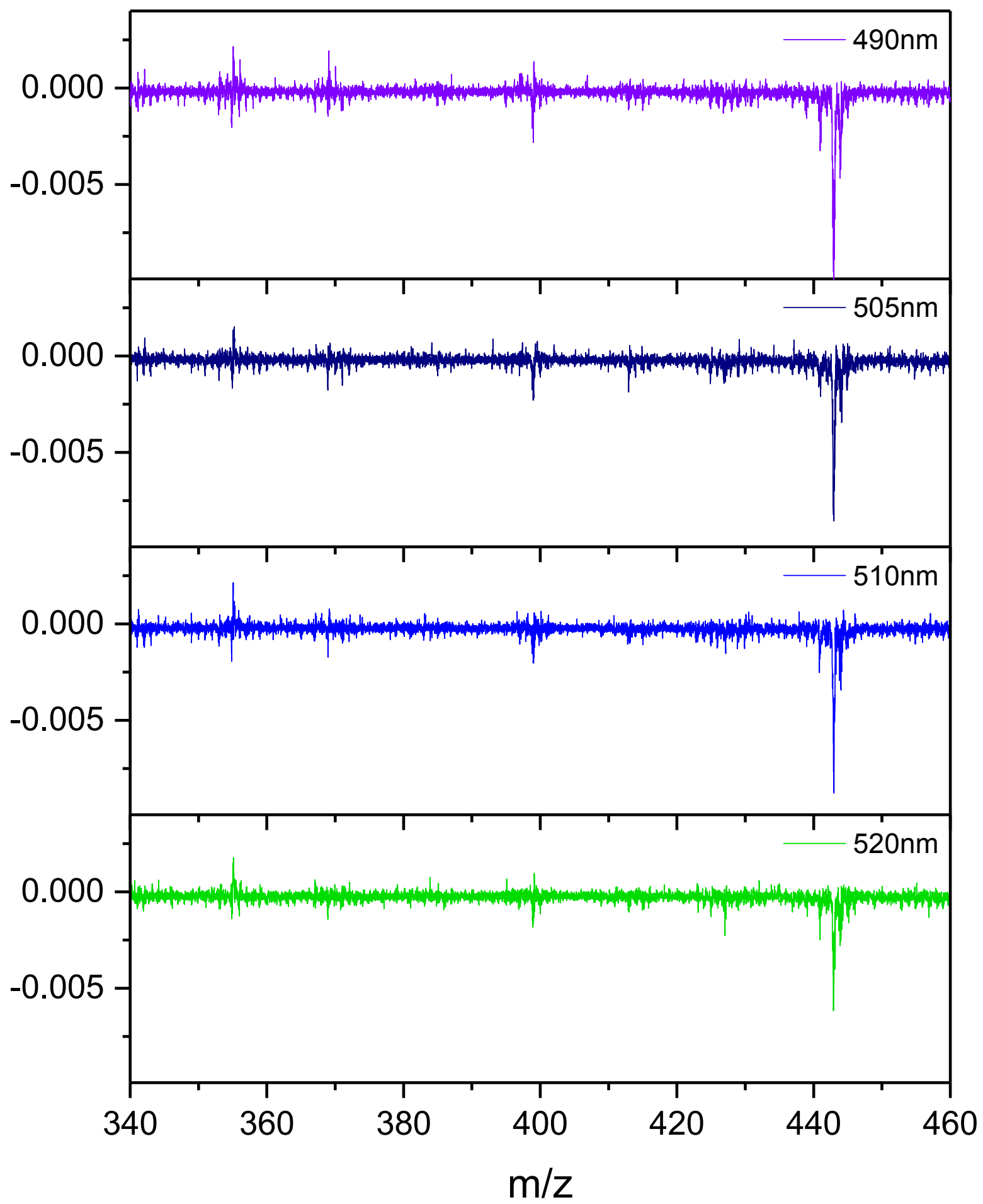

Figure S2. Differential mass spectra of Rhodamine B upon laser irradiation at various wavelengths (480, 500, 520, and 540 nm). Each spectrum was obtained by subtracting the mass spectrum recorded without laser irradiation from that recorded with laser irradiation. Positive peaks indicate fragment ions generated via laser-induced photodissociation, while negative peaks represent the depletion of the parent ion (*m/z* 443) due to photon absorption. The observed photodepletion signals confirm the wavelength-dependent fragmentation efficiency.

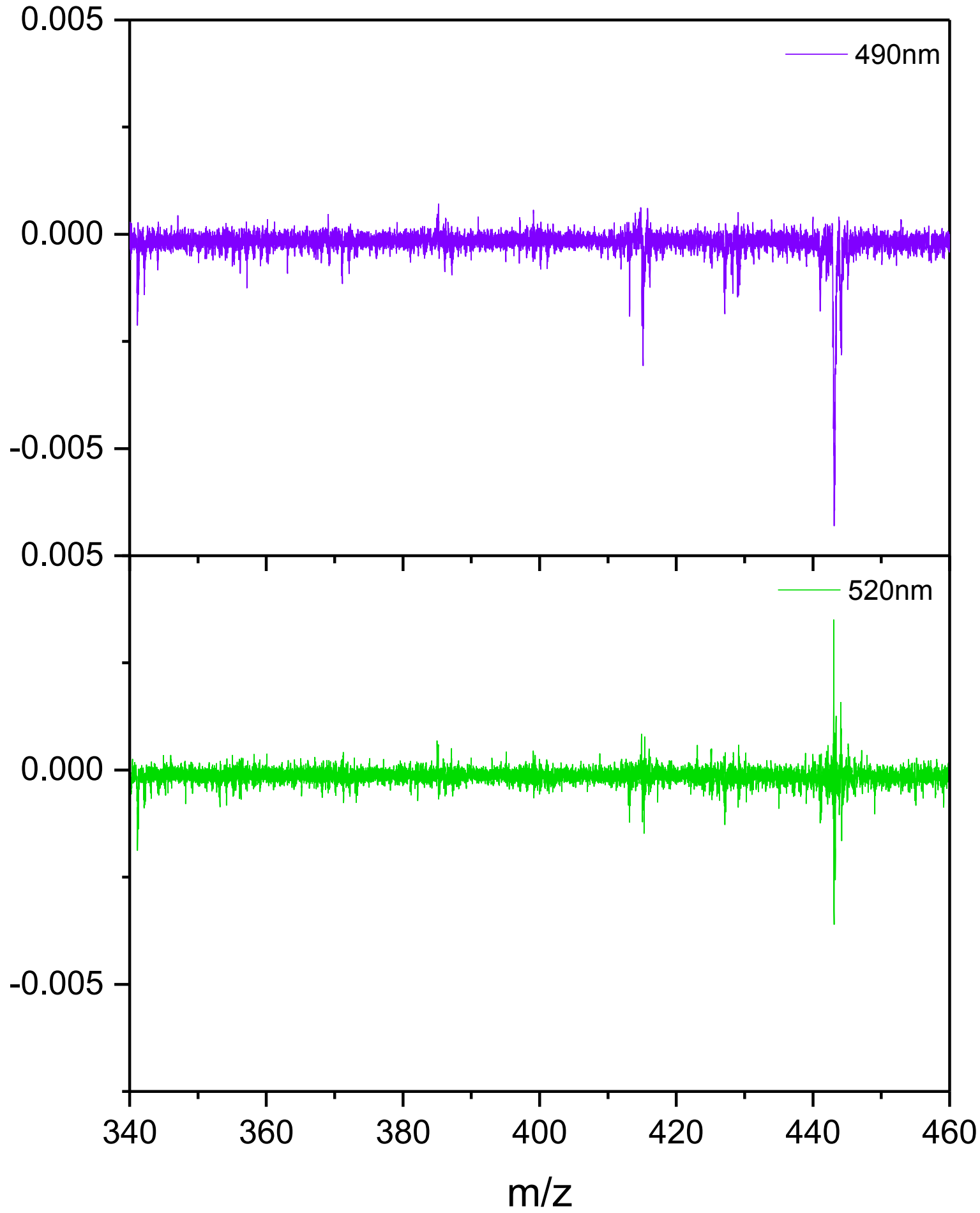

Figure S3. Differential mass spectra of Rhodamine 6G obtained upon laser irradiation at 520 and 540 nm. Each spectrum was generated by subtracting the spectrum recorded without laser irradiation from that recorded with laser irradiation. Positive peaks represent fragment ions newly formed by laser-induced photodissociation, while negative peaks indicate the photodepletion of the parent ion (*m/z* 443) due to photon absorption. Notably, the parent ion signal at 540 nm shows no significant photodepletion, indicating negligible photon absorption at this wavelength. The photodissociation efficiency and fragment ion patterns exhibit clear wavelength dependence, reflecting the distinct photophysical behavior of Rhodamine 6G.

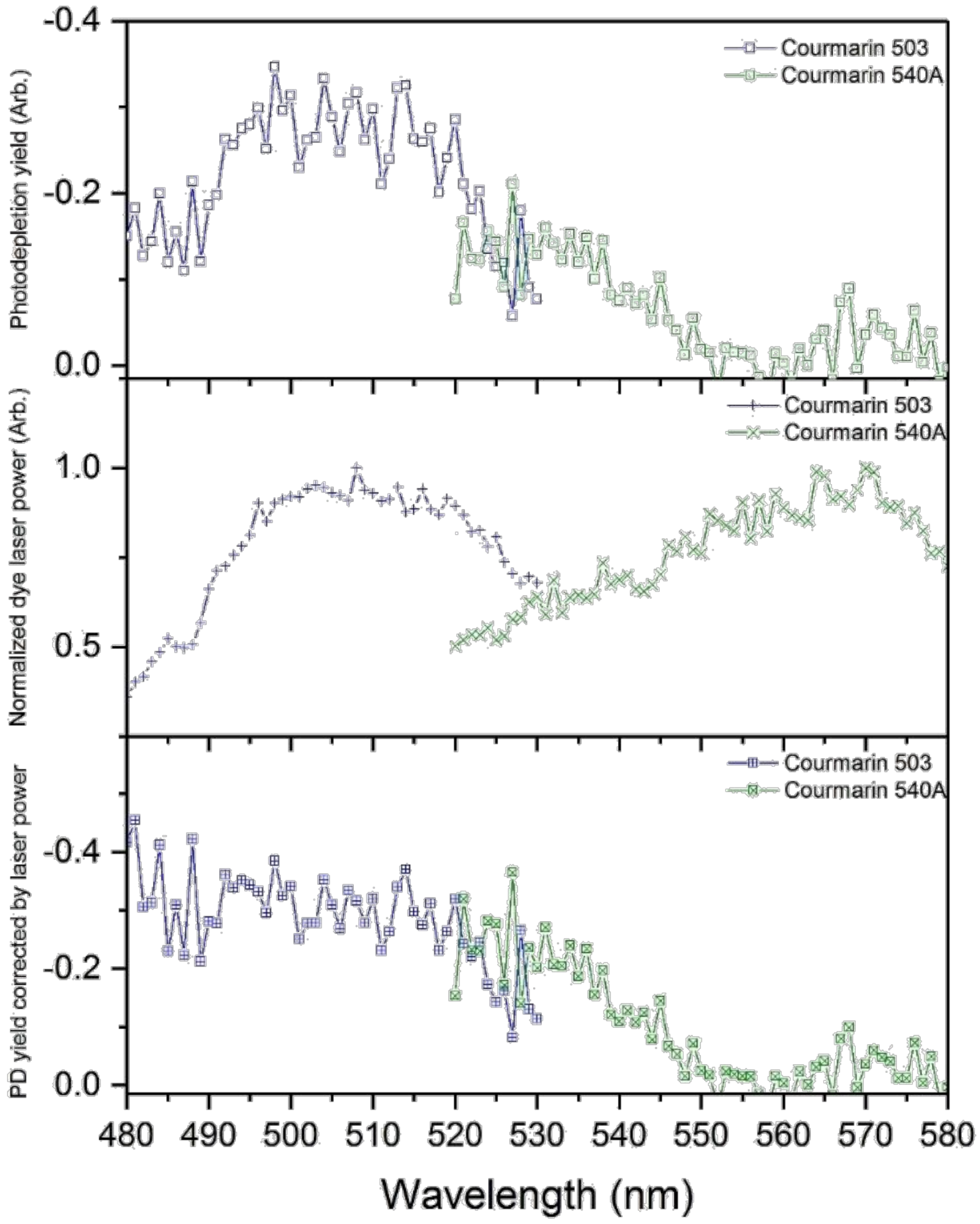

Figure S4. (Top) Photodepletion spectrum recorded for Rhodamine B across the 480–580 nm wavelength range using two laser dyes, namely Coumarin 503 and Coumarin 540A. Negative values represent the depletion of the parent ion (*m/z* 443) upon photon absorption. (Middle) Wavelength-dependent power curves of the dye laser outputs of the different dyes, normalized for comparison. (Bottom) Power-normalized photodepletion spectrum of Rhodamine B obtained across the 480–580 nm range using the Coumarin 503 and Coumarin 540A laser dyes. The photodepletion yield at each wavelength was divided by the corresponding laser power to compensate for output fluctuations, thereby revealing the intrinsic wavelength dependence of the molecular photodissociation efficiency.

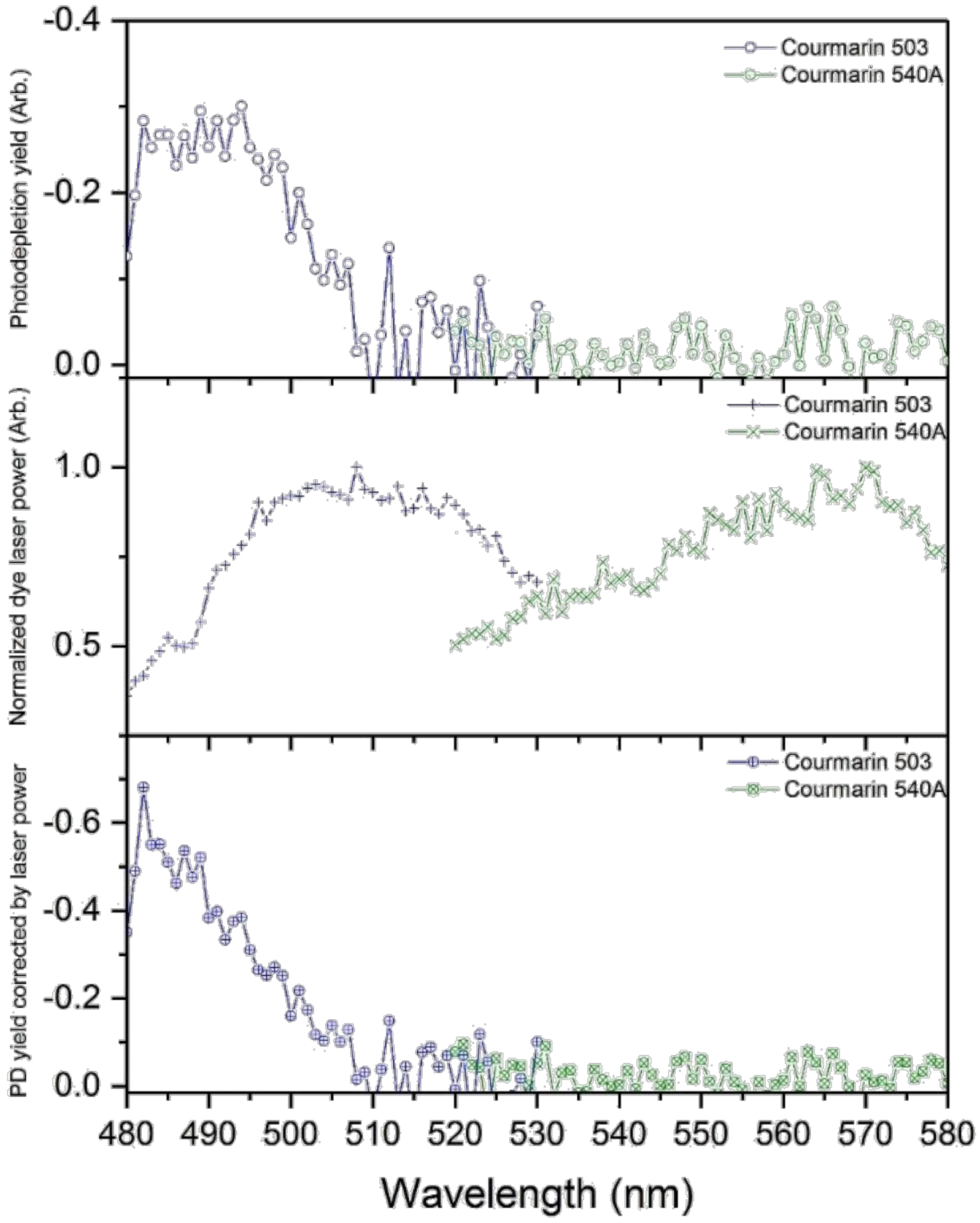

Figure S5. (Top) Photodepletion spectrum recorded for Rhodamine 6G across the 480–580 nm range using two laser dyes, namely Coumarin 503 and Coumarin 540A. The negative signal represents the depletion of the parent ion (*m/z* 443) upon photon absorption at each wavelength. (Middle) Wavelength-dependent output power of the dye laser for each dye, normalized for relative comparison. (Bottom) Power-normalized photodepletion yield of Rhodamine 6G, highlighting the intrinsic photodissociation efficiency as a function of the excitation wavelength. The negligible depletion observed at wavelengths >540 nm indicates minimal photon absorption in this range.
